# Effect of Midazolam Alone Versus Midazolam with Maternal Presence on Pain and Anxiety of Lumbar Puncture in 6 to 24-Month-Old Children

**Published:** 2020

**Authors:** Farzad FERDOSIAN, Reihaneh ESTEGHAMAT, Razieh FALLAH, Tamkin SHAHRAKI

**Affiliations:** 1 Department of Pediatrics, Children Growth Disorder Research Center, Shahid Sadoughi University of Medical Sciences, Yazd, Iran; 2 Student′s Research Center, Shahid Beheshti University of Medical Sciences, Yazd, Iran

**Keywords:** Child, Lumbar Puncture, Maternal Presence, Pain, Midazolam, Sedation

## Abstract

**Objectives:**

Midazolam at a dosage of 0.5 mg/kg induces anxiolytic effects in 90% of children. This study was performed to elucidate whether intravenous midazolam with maternal presence is more efficient than intravenous midazolam alone in the reduction of pain and anxiety of lumbar puncture (LP) in 6 to 24-month-old children.

**Materials & Methods:**

In this not-blinded clinical trial, we included 60 children aged 6 to 24 months old undergoing LP in the Pediatric Ward of Shahid Sadoughi Hospital, Yazd, Iran, from September 2014 to March 2015. The participants were randomly assigned to two groups, and all of them received painless injection of 0.5 mg/kg midazolam five minutes before LP. In group I, LP was performed with maternal presence and in group II, the mothers were absent. The primary outcomes included anxiety and pain scores before LP and during needle insertion to the skin for LP. The secondary outcomes comprised of success rates in the reduction of anxiety (anxiety score of four and more) and pain (pain score of less than three) when the needle was inserted to the skin for LP.

**Results:**

Twenty-eight girls and 32 boys were evaluated in the two groups. Maternal presence was found to be effective in the reduction of anxiety (2.7±0.65 vs. 3.83±0.87; P=0.001) and pain scores (3.8±1.75 vs. 6.1±1.63, P=0.001). In the maternal presence group, success rate in anxiety (76.7% vs. 16.6%; P=0.0001) and pain reduction (63.3 % vs. 6.7%; P=0.0001) was higher than in the midazolam alone group.

**Conclusion:**

Maternal presence during lumbar puncture can reduce pain and anxiety among 6 to 24-month-old children.

## Introduction

Untreated pain can negatively affect the development of the central nervous system (CNS), which has long-standing psychological consequences (1). Lumbar puncture (LP) is a painful diagnostic procedure which can help in the detection of CNS infections, subarachnoid hemorrhage, brain tumors and demyelinating-degenerative disorders (2). 

Many medications and non-pharmacological methods or combination of drugs with psychological interventions have been implemented to assuage anxiety and pain in children. Providing a safe and efficient sedative-analgesic program in children needs collaboration among physicians, children and parents (3). 

Most diagnostic and therapeutic procedures performed in infants and children are without analgesia (4). Although the use of sedatives before LP lowers the incidence of traumatic lumbar puncture (5), Fein et al. study showed that pain management strategies including local anesthesia, sedation, or combination of both before LP have been used only in 23.8% of children. Thus, they concluded that suitable pain control programs should be implemented before LP in children and infants. (6) 

Midazolam is a medication with rapid onset of action and little adverse events, which can be used via oral, intravenous, intranasal and intramuscular routes of administration, and it has been used as a sedative before medical procedures for many years. Cochrane Database systematic review showed that intravenous midazolam in comparison with placebo decreased the anxiety of diagnostic and therapeutic medical procedures. (7) 

Midazolam is a short-acting, potent, hypnotic and anxiolytic drug most commonly used in pediatric anesthesia and induces anxiolytic effects in 90% of children at a dosage of 0.5 mg/kg. (8) Different procedural sedation regimens have been used in different pediatric departments and we use 0.5 g/kg intravenous midazolam for sedation before LP in our department.

Up until mid-1990s, parental presence during invasive procedures was not adhered to or widely accepted (9), and medical staff, especially nurses disagreed to paternal presence during medical invasive procedures (9, 10). Nonetheless, nurses believe that the presence of family reduces children’s distress and parental anxiety. (10) 

A literature review by Boudreaux et al. showed that parents of sick children mostly opted to stay during aggressive medical procedures. Parents who stayed during the procedure commonly described desirable experiences and felt that it was useful for their sick children and the authors recommended performing further randomized clinical trials to evaluate the beneficial effects of paternal presence during invasive procedures. (11) Based on the evidence, family presence is useful for sick children, their parents and healthcare providers. As the presence of family is becoming more widely accepted, medical staff should prepare parents of sick children and identify the impediments to this practice. (12) On the other hand, the combination of non-pharmacological pain management methods and pharmacologic medications in newborns might be more efficient in pain control and most often a combination of them is used in practice. (13) 

Maternal presence during invasive medical procedures may increase anxiety in mothers; therefore, in pediatric wards of Iran, parents should not be present during invasive procedures. 

The effect of parental presence during invasive medical procedures has not been evaluated in Iran, while it is an important challenge in pediatric wards of this country. Thus, we sought to evaluate the efficacy of the combination of a non-pharmacological pain management method (maternal presence) and intravenous midazolam as a pharmacological intervention before lumbar puncture. This clinical trial was carried out to elucidate whether intravenous midazolam with maternal presence is more efficient than intravenous midazolam alone in the reduction of pain and anxiety of lumbar puncture in 6 to 24-month-old children.

## Materials & methods

In this randomized, not-blinded, parallel-group clinical trial, we enrolled all the consecutive 6 to 24-month-old children who were admitted to the Pediatric Ward of Shahid Sadoughi Hospital, Yazd, Iran, from March to June 2015. The participants were candidates for LP based on the clinical judgment of pediatricians.

Based on Z formula and with a confidence interval of 95% , power of 80%, type one error of 5%, success in anxiety reduction (obtaining anxiety score of more than 4 during LP) of 52% for maternal presence in our pilot study and an effect size (difference in frequency of success in anxiety reduction between the two groups) of 30% for this primary outcome, the sample size was calculated to be 30 children in each group.

The inclusion criteria comprised of children aged 6-24 months, with the American Society of Anesthesiologists (ASA) physical status I-II, undergoing lumbar puncture based on the clinical judgment of a pediatrician and not having received sedative hypnotic drugs or systemic analgesic drugs (acetaminophen or ibuprofen) within the past 48 hours.

The exclusion criteria consisted of neurodevelopmental delay or mental retardation, loss of consciousness (Glasgow coma scale less than 12), symptoms of increased intracranial pressure and having undergone lumbar puncture more than twice.

The developmental status of the children was assessed using the Denver II Developmental Screening Test. We used computer-generated equal simple randomization by random numbers, and the allocation ratio was 1:1 for the two groups.

Since the mothers were present during LP in the maternal presence group and the resident of research who assessed the primary and secondary outcomes and gathered the data and the resident of research who did LP were seeing the mothers, blinding of the participating mothers, pediatric resident, data collector and outcome assessor was not possible, and only the data analysts were kept blinded to the allocation. However, concealment was performed by placing the group number for each serially participating child in a numbered and sealed opaque envelope which was opened by the pediatric neurologist immediately before LP. Randomization and concealment were done by a researcher with no clinical involvement in the trial. 

**Table.1:** Comparison of some characteristics of children in both groups

Groups		Midazolam alone		Midazolam with maternal presence		P. Value	
Data							
Sex	Girl		17		11		0.1
	Boy		13		19		
Age in year (mean ±SD)		1.13 ± 0.48		1.12 ± 0.39		0.3	
Weight in kg (mean ±SD)		9.13±3.29		9.7±2.81		0.5	
Anxiety score before lumbar puncture (mean ±SD)		1.15 ± 0.51		1.64 ± 0.21		0.4	
Pain score before lumbar puncture (mean ±SD)		1.01 ± 0.12		1.21 ± 0.05		0.7	

**Table.2:** Comparison of anxiety and pain scores during lumbar puncture (LP) and the frequency of traumatic LP in the two groups

Groups		Midazolam alone	Midazolam with maternal presence	P. Value
Data				
Anxiety score during lumbar puncture (mean ±SD)		3.83 ± 0.87	2.7 ± 0.65	0.001
Pain score during lumbar puncture (mean ±SD)		6.1 ± 1.63	3.8 ± 1.75	0.001
Traumatic lumbar puncture	Yes	2	3	0.6
	No	28	27	

**Table 3:** Comparison of success rates in the reduction of anxiety and pain during needle insertion for lumbar puncture in both groups

Groups		Midazolam alone	Midazolam with maternal presence	P. Value
Data				
Success in anxiety reduction	Yes	5	23	0.0001
	No	25	7	
Success in pain reduction	Yes	2	19	0.0001
	No	28	11	

In both groups, 0.5 mg/kg midazolam was injected intravenously five minutes before LP and the children were randomly assigned to the two groups. In group I, lumbar puncture was performed with maternal presence, and in group II, the mothers were out of the room during LP of their children.

The midazolam used in the research was 5 mg/ml vial from Aburaihan Co. Tehran, Iran, and in all the children, midazolam was injected under similar conditions, by similar needles and by a trained pediatric ward nurse. All the lumbar punctures were performed by one expert pediatric resident in the lateral decubitus position. In the maternal presence group, during lumbar puncture of the children, the mothers were in the room and sat face to face with their children, held the child^’^s hand and dandled it and the resident who performed LP was behind the child.

Primary and secondary outcomes were assessed by the pediatric resident. The primary outcomes included baseline anxiety and pain scores before skin needle insertion and anxiety and pain scores while the needle was being inserted to the skin for LP. The secondary outcomes included success rate in reducing anxiety during skin needle insertion (anxiety score of four and more) and success rate in reducing pain when the needle was inserted to the skin for LP (pain score of less than three).

Anxiety score was evaluated by Anxiety score (14) and pain score was assessed based on the modified Children's Hospital of Eastern Ontario Pain Scale (CHEOPS) (15). 

An anxiety score of four or more during needle insertion for LP was considered as success in reducing anxiety and obtaining a pain score of less than three based on the CHEOPS during needle insertion was considered as success in pain reduction.

Data were analyzed using SPSS version 17. The recorded data were assessed for normal distribution using the Kolmogorov-Smirnov test, and Chi-square test was used for the analysis of categorical variables. Also, continuous variables and means were compared between the two groups using independent *t*-test. Differences were considered significant at *P*-value less than 0.05.

Informed consent was obtained from parents of the children before enrollment and the study was approved by the Ethics Committee of Shahid Sadoughi University of Medical Sciences, Yazd, Iran. After enrollment of the children, the researcher asked the mothers about their willingness to be present in the lumbar puncture room in the maternal presence group, and the child was excluded if the mother did not want to be present during LP of her child. 

## Results

The design and conduct of this trial were straightforward, and we did not have any losses or exclusions from the analysis. Overall, 28 girls and 32 boys with the mean age of 1.06 ± 0.43 year were evaluated in the two groups. Based on the Kolmogorov-Smirnov test, the data had normal distribution.

Comparison of some characteristics of the children in the two groups is presented in Table 1, which indicates that no significant differences were observed in terms of gender distribution, mean age, mean weight, mean anxiety or pain score before LP.

Table 2 shows the comparison of anxiety and pain scores during LP and the frequency of traumatic lumbar puncture in the two groups, which indicates that maternal presence was effective in the reduction of anxiety and pain scores of the children during LP, but the frequency of traumatic LP was not significantly different between the groups.

Comparison of frequencies of success rate in reducing anxiety (obtaining an anxiety score of four and more) and pain (pain score of less than three) during skin needle insertion for LP is presented in Table 3. This table demonstrates that in the maternal presence group, the success rate in anxiety and pain reduction was more than in the midazolam alone group.

## Discussion

Parental presence during invasive procedures might decrease sick children’s distress and parental anxiety (10), and it is one of the challenges faced in pediatric departments; thus, parents’ tendency to attend the procedure should be considered. In a study in Madrid, Spain, 66.3% of parents tended to be present for LP (15) and it is logical to evaluate the beneficial effects of parental presence during invasive procedures by randomized clinical trials. (11)

The results of the present randomized clinical trial showed that the combination of maternal presence as a non-pharmacologic pain management strategy and intravenous midazolam that was injected five minutes before LP was more efficient than intravenous midazolam alone in the reduction of pain and anxiety of LP among 6 to 24-month-old children.

In a randomized clinical trial performed in Greece, the efficacy of parental presence and attention distraction of the child by a toy in the reduction of pain and anxiety of children undergoing a painful procedure was compared with a control group. Parental presence was more effective in the reduction of respiratory rate, blood pressure, pulse rate, pain verbal rating scale score and anxiety score of children. (16) 

Cochrane Database Systematic Review of Manyande et al. showed that parental presence at induction of anesthesia did not reduce children’s anxiety during induction. (17) In a systematic review, Piira et al. evaluated the effects of parental presence in the treatment of 1256 children and concluded that although the presence of parents may not have an obvious impact on behavioral consequences, their presence is a potential advantage for them. (18) 

In the present study, maternal presence did not increase the rate of traumatic LP, which is compatible with the results of a previous study that indicated family presence did not increase the risk of traumatic or unobtainable LP. (19)

The efficacy of family presence during resuscitation and invasive medical procedures was evaluated by Mangurten et al. (20) Care of sick children was not disturbed by paternal presence and parents who were affirmative about their presence thought that it assisted their kids and assumed that it alleviated their anxiety. The parents reported an active role during the event, and most of them stated that they were rightful to be present. None of the parents reported traumatic memories after three months. (20) 

In order to implement pediatric departments’ parental presence protocol, first it is essential to identify the positive effects of family presence (21) to accept the presence of parents during invasive procedures. Then, parents should be given the option to be present during invasive medical procedure of their sick children. (18) 

**In conclusion**, based on the results of this randomized clinical trial, the combination of maternal presence during LP with intravenous midazolam could reduce pain and anxiety of lumbar puncture in 6 to 24-month-old children.

## References

[1] Mahajan C, Dash HH (2014). Procedural sedation and analgesia in pediatric patients. J Pediatr Neurosci.

[2] Schulga P, Grattan R, Napier C, Babiker MO (2015). How to use… lumbar puncture in children. Arch Dis Child Educ Pract Ed.

[3] Krieser D, Kochar A (2016). Paediatric procedural sedation within the emergency department. J Paediatr Child Health.

[4] Gorchynski J, McLaughlin T (2011). The routine utilization of procedural pain management for pediatric lumbar punctures: are we there yet?. J Clin Med Res.

[5] Glatstein MM, Zucker-Toledano M, Arik A, Scolnik D, Oren A, Reif S (2011). Incidence of traumatic lumbar puncture: experience of a large, tertiary care pediatric hospital. Clin Pediatr (Phila).

[6] Fein D, Avner JR, Khine H (2010). Pattern of pain management during lumbar puncture in children. Pediatr Emerg Care.

[7] Conway A, Rolley J, Sutherland JR (2016). Midazolam for sedation before procedures. Cochrane Database Syst Rev..

[8] Wetzel RC, Kliegman RM, Stanton BF, Schor NF, St Geme JW, Behrman RE (2016). Anesthesia , peerioperative care and sedation. Nelson Textbook of Pediatrics.

[9] Prati G, Monti M (2010). Family presence during cardiopulmonary resuscitation and other invasive procedures. G Ital Med Lav Ergon.

[10] Corniero P, Gamell A, Parra Cotanda C, Trenchs V, Cubells CL (2011). Family presence during invasive procedures at the emergency department: what is the opinion of Spanish medical staff?. Pediatr Emerg Care.

[11] Boudreaux ED, Francis JL, Loyacano T (2002). Family presence during invasive procedures and resuscitations in the emergency department: a critical review and suggestions for future research. Ann Emerg Med.

[12] Duran CR, Oman KS, Abel JJ, Koziel VM, Szymanski D (2007). Attitudes toward and beliefs about family presence: a survey of healthcare providers, patients' families, and patients. Am J Crit Care.

[13] Krasteva M (2013). Pain in the neonatal period II Non-pharmacological and pharmacological treatment. Akush Ginekol (Sofiia).

[14] Lee YS, Kim WY, Choi JH, Son JH, Kim JH, Park YC (2010). The effect of ketamine on the incidence of emergence agitation in children undergoing tonsillectomy and adenoidectomy under sevoflurane general anesthesia. Korean J Anesthesiol.

[15] Pérez Alonso V, Gómez Sáez F, González-Granado LI, Rojo Conejo P (2009). Presence of parents in the emergency room during invasive procedures: do they prefer to be present?. An Pediatr (Barc).

[16] Matziou V, Chrysostomou A, Vlahioti E, Perdikaris P (2013). Parental presence and distraction during painful childhood procedures. Br J Nurs.

[17] Manyande A, Cyna AM, Yip P, Chooi C, Middleton P (2015). Non-pharmacological interventions for assisting the induction of anaesthesia in children. Cochrane Database Syst Rev.

[18] Piira T, Sugiura T, Champion GD, Donnelly N, Cole AS (2005). The role of parental presence in the context of children's medical procedures: a systematic review. Child Care Health Dev.

[19] Nigrovic LE, McQueen AA, Neuman MI (2007). Lumbar puncture success rate is not influenced by family-member presence. Pediatrics.

[20] Mangurten J, Scott SH, Guzzetta CE, Clark AP, Vinson L, Sperry J, Hicks B, Voelmeck W (2006). Effects of family presence during resuscitation and invasive procedures in a pediatric emergency department. J Emerg Nurs.

[21] Mekitarian FF, Angelo M (2015). Family's presence in the pediatric emergency room: opinion of health's professionals. Rev Paul Pediatr.

